# Assessment of erythrocyte alloimmunization among patients treated at a Brazilian university hospital

**DOI:** 10.1016/j.htct.2024.04.128

**Published:** 2024-09-07

**Authors:** Higor Silva Contelli, Mário Cézar de Oliveira, Aline Akemi Segatti Ido, Elaine Machado Francalanci, Patrícia Oliveira da Cunha Terra, Elmiro Ribeiro Filho, Deivid William da Fonseca Batistão, Sabrina Royer

**Affiliations:** a Instituto de Ciências Biomédicas (ICBIM), Universidade Federal de Uberlândia (UFU), Uberlândia, MG, Brazil; b Agência Transfusional (AGETRA), Hospital de Clínicas da Universidade Federal de Uberlândia (HCUFU/EBSERH), Uberlândia, MG, Brazil; c Hemocentro Regional de Uberlândia, Uberlândia, MG, Brazil; d Faculdade de Medicina (FAMED), Universidade Federal de Uberlândia (UFU), Uberlândia, MG, Brazil

**Keywords:** Erythrocyte transfusion, Antigen-antibody reactions, Blood group incompatibility, Blood group antigens, Transfusion reaction

## Abstract

**Introduction:**

Alloimmunization and transfusion reactions underscore the crucial role of precise immunohematological techniques to enhance safety in transfusion. This study aims to determine the frequency of alloimmunization in patients treated at a Brazilian university hospital, investigate demographic, clinical, and epidemiological characteristics of patients with positive irregular antibody screening, as well as to assess the frequency of erythrocyte antigens and anti-erythrocyte antibodies in the population.

**Materials and methods:**

This retrospective observational study included all irregular antibody-positive patients treated at the transfusion service of Hospital de Clínicas of the Federal University of Uberlandia between January 2019 and December 2020.

**Results:**

Of the 201 irregular antibody-positive patients, alloimmunization was more common in women (64.2%) than in men (35.8%). Blood groups A (39.8%) and O (38.8%), and Rh positive samples (69.1%) predominated, and about half (48.2%) of the patients were transfused for preoperative procedures. The most frequently found clinically significant alloantibodies were anti-D (27.2%), anti-E (15.0%), and anti-Kell (11.5%). Of the patients, 30.6% had multiple antibody associations, with anti-D and anti-C being the most common combination. Erythrocyte immunophenotyping was performed for 76 patients with the most frequent antigens detected being e (100%), c (86.8%), and C (40.8%). Among the 14 pregnant women evaluated, most were multiparous, 85.7% had anti-D as the most prevalent antibody, and had the A-negative blood type (33.3%).

**Conclusion:**

Alloantibody screening and identification associated with erythrocyte immunophenotyping are necessary for a better understanding of the alloimmunized population, ensuring greater safety and efficacy of transfusion therapy in the hospital setting.

## Introduction

Transfusion Medicine has evolved becoming increasingly safer for patients requiring therapy with blood components and hemoderivatives.^1^^,^^2^ The first successful blood transfusion in animals was performed by physician Richard Lower in 1666. However, it was only after the discovery of blood types by Karl Landsteiner at the beginning of the 20th century that the first blood transfusion preceded by tests of compatibility using the ABO system could be performed. This was carried out by Reuben Ottenberg in 1907. Despite this historical breakthrough, blood transfusion continued to be considered a risky procedure.^2^ The need to treat wounded soldiers who died of acute bleeding in the two world wars intensified research on the subject, and blood transfusion began to be used on a large scale after that period. This culminated in the creation of transfusion commissions aimed at ensuring the correct use of hemotherapy.^2^ With the development of transfusion medicine as a medical specialty, blood processing has undergone technological innovations. Today, good practices and surveillance ensure safety throughout the blood cycle, providing safe hemotherapy for patients.^2^

To enable individual screening of blood components, it is necessary not only to consider the interaction between donor antigens and recipient antibodies, but also to select the most appropriate blood component based on the patient's clinical condition and individual characteristics.^3^ Both assessments aim to detect compatibility through cross-reactivity and minimize the risk of adverse reactions.^4^ According to Brazilian regulations, the pre-transfusion tests that must be performed include ABO and Rh typing, irregular antibody screening (IAS), and crossmatching.^3^ These tests are performed to determine the patient's erythrocyte profile and select the most suitable blood component.^3^

Exposure to antigens, not only of the ABO and Rh systems but also of several other systems present in the erythrocyte membrane, all of which are catalogued in 45 blood systems, may result in alloimmunization.^5^ When the body is exposed to non-self-antigens, the immune system responds by activating B lymphocytes, leading to the production of antibodies that neutralize these antigens.^6^ The risk of developing alloantibodies depends on factors such as the number and frequency of transfusions, pregnancy, antigen immunogenicity, recipient immune response, patient's ethnicity, and differences in the pattern of antigens, both of the donor and of the recipient.^6^^,^^7^ Alloimmunization predisposes patients to acute or delayed hemolytic transfusion reactions, in addition to making the selection of compatible blood components difficult.^7^^,^^8^

The need to minimize transfusion reactions has led to the adoption of increasingly precise immunohematological techniques in transfusion practices, as well as the optimization of strategies to prevent alloimmunization. The latter requires an understanding of the characteristics of the alloimmunized population. Therefore, the objectives of this study were to determine the frequency of alloimmunization among patients evaluated during the study period, investigate the demographic, clinical, and epidemiological characteristics of patients with a positive result in the IAS test, and to assess the frequency of erythrocyte antigens and anti-erythrocyte alloantibodies in the analyzed population.

## Material and methods

### Hospital settings

This study was conducted at the Hematology and Hemotherapy Unit - Transfusion service (AGETRA) of Hospital de Clínicas of the Federal University of Uberlândia (HC-UFU/EBSERH). This is a public, academic, tertiary care hospital complex with a capacity for 525 patients. It serves as a referral center for an estimated population of over two million residents of Uberlândia and 81 municipalities in the Triângulo Mineiro and Alto Paranaíba regions. All hospital services are provided under the Brazilian national healthcare service (SUS).

### Study design

A retrospective observational study was conducted, including all patients who tested positive for IAS that were treated in the transfusion service from January 2019 to December 2020. Patients with a first-time positive IAS were included in the study, while those who presented with only autoantibodies were excluded. Demographic, clinical, and epidemiological variables were obtained through the analysis of medical records and the AGETRA database with an individual form being completed with patient data. The information was tabulated using Microsoft Excel (Microsoft Corporation, Washington).

### Data analysis

Descriptive analysis was utilized to characterize demographic and clinical data, and figures were generated using GraphPad Prism 9.0® software (San Diego, CA).

### Pre-transfusional tests

Following Brazilian federal regulations,^3^ the pre-transfusion tests performed by AGETRA included recipient ABO/RhD classification, search for IAS in the recipient using the microtube technique with ID-DiaCell I and II reagents (Diamed-Biorad®), ABO/RhD reclassification of the selected blood bag, and cross-matching. In cases of positive IAS, the specific identification of alloantibodies was carried out by the Hemominas Foundation/Belo Horizonte. Patients with oncohematological diseases (leukemia and myelodysplastic syndrome), hemoglobinopathies (sickle cell anemia and thalassemia), chronic kidney disease (CKD), or those receiving multiple transfusions, were also subjected to erythrocyte immunophenotyping (Diamed-Biorad®). Unusual agglutination profiles were reported as ‘unidentified antibodies’.

### Ethical considerations

This project was approved by the research ethics committee of the Federal University of Uberlândia (CAAE 42796620.5.0000.5152).

## Results

**Figure 1:**
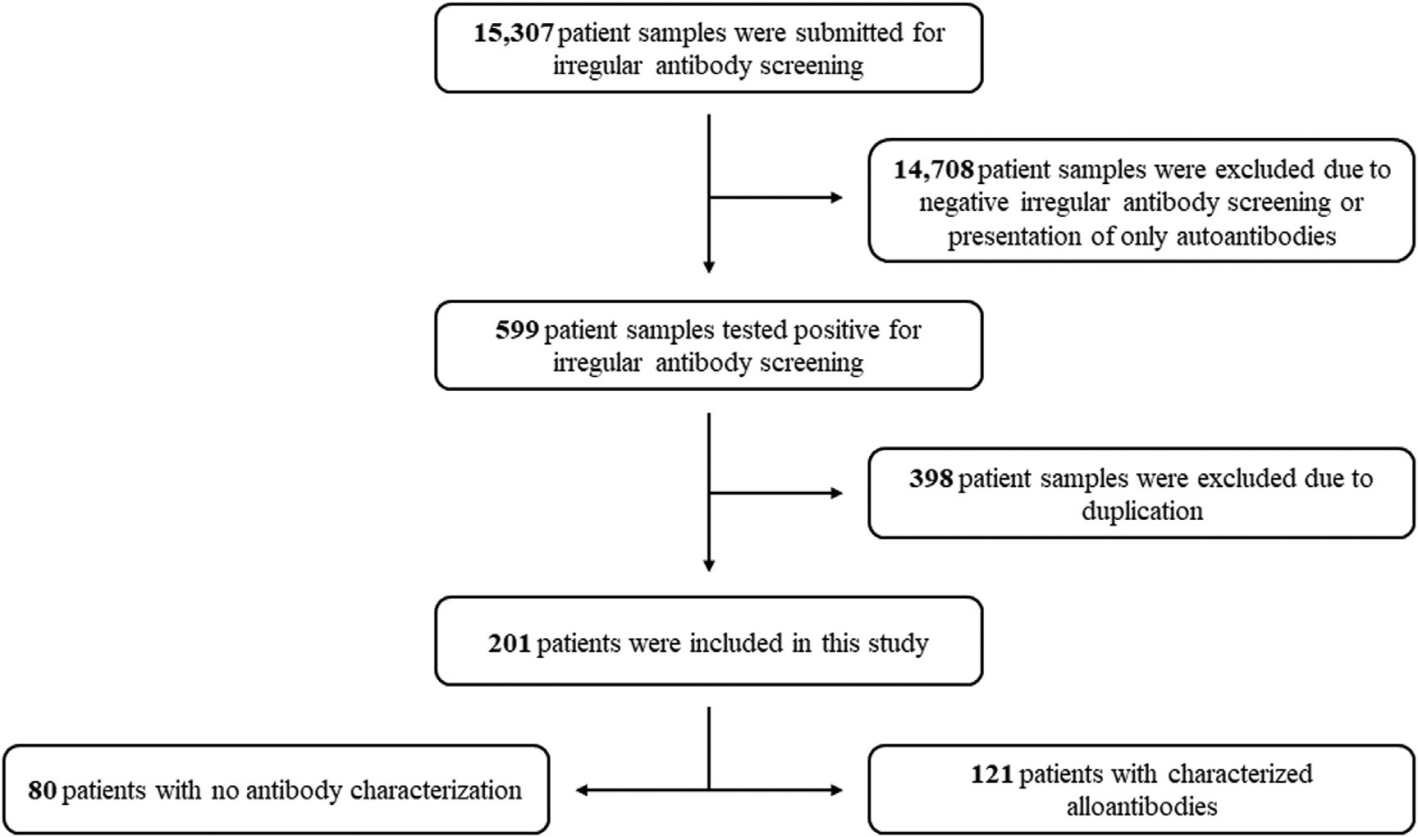
Flow diagram of the patients analyzed in the study.

Between January 1 2019 and December 31 2020, 15,307 patient samples underwent IAS at AGETRA (HC-UFU/EBSERH). Among these, 599 samples tested positive for IAS, constituting 3.9% of the total samples. After excluding duplicate samples (n = 398), the study focused on 201 patients with confirmed positive IAS results out of 14,909 patients attended by AGETRA during the specified period. The prevalence of positive IAS samples in the unit averaged 1.3% (201/14,909 - Figure 1).

The median age of the 201 patients was 53 years (interquartile range [IQR]: 32-65 years), with alloimmunization occurring more frequently in women (64.2%) than in men (35.8%). Blood groups A (39.8%) and O (38.8%), and RhD positive samples (69.1%) were the most common. Approximately half (48.2%) of the patients were transfused due to preoperative procedures. During the hospitalization period, 32.8% of the patients received 1-3 transfusions, and 71.6% had a history of transfusions (Table 1).

**Table 1:** Clinical and epidemiological characteristics of irregular antibody-positive patients evaluated by the transfusion service (AGETRA) of Hospital de Clínicas of the Federal University of Uberlândia (HC-UFU/EBSERH) from January 2019 to December 2020.

Characteristic	n = 201 (%)
**Gender – n (%)**	
Male	72 (35.8)
Female	129 (64.2)
**Age – median (IQR)**	53 (32-65)
**Ethnicity – n (%)**	
White	101 (50.2)
Others	100 (49.7)
**ABO typing – n (%)**	
A	80 (39.8)
B	28 (13.9)
AB	12 (6.0)
O	78 (38.8)
Indefinite	3 (1.5)
**RhD typing – n (%)**	
Positive	139 (69.1)
Negative	59 (29.3)
Indefinite	3 (1.5)
**Clinical indication for transfusion – n (%)**	
Preoperative	97 (48.2)
Anemia	9 (4.5)
Pregnancy/Birth	18 (9.0)
Kidney or Hearth Disease	17 (8.3)
Hematologic disease	20 (10.0)
Malignancy^a^	40 (20.0)
**Number of transfusions received during hospitalization – n (%)**	
>10	12 (6.0)
4 to 10	24 (12.0)
1 to 3	66 (32.8)
0	99 (49.2)
**Transfusion history – n (%)**	
Yes	144 (71.6)
No	50 (24.9)
Uninformed	7 (3.5)
**Previous surgery – n (%)**	
Yes	115 (57.2)
No	77 (38.3)
Uninformed	9 (4.5)
**Length of stay, in days – median (IQR)**	8.5 (3-21)
**Outcome 30 days after last transfusion – n (%)**	
Discharge	171 (85.1)
Death	30 (14.9)

IQR: Interquartile range.

a Solid tumor and/or oncohematological diseases.

**Figure 2:**
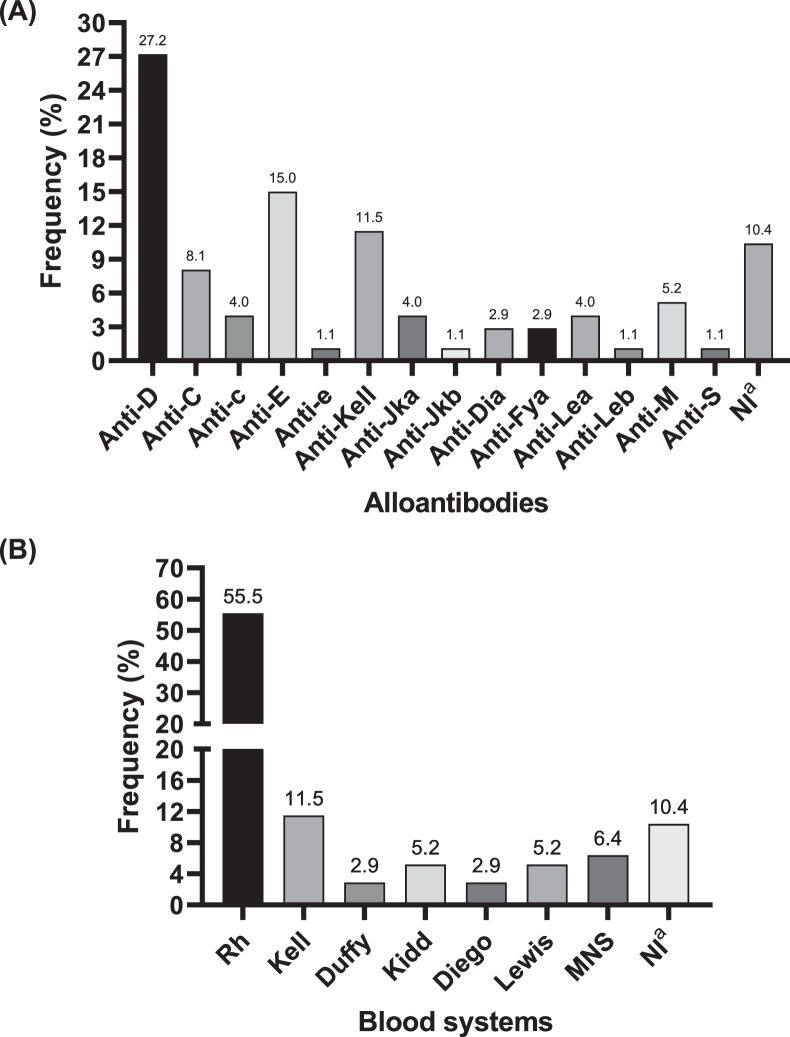
Frequency of alloantibodies detected in 121 of 201 irregular antibody-positive patients evaluated by the transfusion service (AGETRA) of Hospital de Clínicas of the Federal University of Uberlândia (HC-UFU/EBSERH), from January 2019 to December 2020 according to the type of alloantibody (A) and the blood system (B). ^a^Antibodies not identified by the RBC panel used.

Out of the 201 alloimmunized patients identified in AGETRA, 121 had their alloantibodies characterized by the Hemominas Foundation. The remaining 80 patients did not undergo alloantibody characterization due to non-reactivity against the red blood cell panel employed by the reference laboratory, a situation that arises when the reactivity of the test conducted by AGETRA is low, prompting the need for sample confirmation. Another contributing factor was the impossibility to recollect samples by the healthcare team for submission to the reference laboratory. Among the clinically significant alloantibodies characterized, the most frequently found were those belonging to the Rh and Kell blood systems, with prevailing anti-D (27.2%), anti-E (15.0%), and anti-Kell (11.5%) antibodies (Figure 2A and 2B).

Of 201 patients, 121 were confirmed IAS-positive by the Hemominas Foundation, 30.6% (37/121) had profiles with multiple associations of alloantibodies, as shown in Table 2. The association of anti-D and anti-C was the most frequent (Profile 1), followed by anti-c and anti-E (Profile 2) and Anti-C, Anti-Kell and unidentified (Profile 3). Several other associations were identified, but with single occurrences in the studied population.

**Table 2:** Characterization of profiles with multiple antibody associations detected in irregular antibody-positive patients submitted to screening by the Hemominas Foundation, attended at the transfusion service (AGETRA) of Hospital de Clínicas of the Federal University of Uberlândia (HC-UFU/EBSERH), in the period from January 2019 to December 2020.

Profile	n	Alloantibody association
1	8	Anti-D; anti-C
2	3	Anti-c; Anti-E
3	2	Anti-C; Anti-Kell; NI^a^
4	1	Anti-C; Anti-E; Anti-Kell; Anti-Jka
5	1	Anti-E; Anti-Lea; Anti-Leb; NI^a^
6	1	Anti-E; Anti-Kell; Anti-Jkb
7	1	Anti-E; Anti-Kell; Anti-Fya
8	1	Anti-Fya; Anti-S; Anti-M
9	1	Anti-C; Anti-E; Anti-M
10	1	Anti-D; Anti-C; Anti-E
11	1	Anti-D; Anti-C; NI^a^
12	1	Anti-Lea; Anti-Leb
13	1	Anti-Fya; Anti-Lea
14	1	Anti-E; Anti-Kell
15	1	Anti-Jkb; Anti-M
16	1	Anti-e; Anti-Kell
17	1	Anti-E; Anti-Jka
18	1	Anti-E; Anti-Lea
19	1	Anti-D; Anti-Dia
20	1	Anti-E; Anti-Dia
21	1	Anti-C; Anti-e
22	1	Anti-E; Anti-S
23	1	Anti-E; NI^a^
24	1	Anti-C; NI^a^
25	1	Anti-D; NI^a^
26	1	Anti-E-NI^a^
27	1	Anti-Jka; N^a^

a Antibodies not identified by the RBC panel used.

According to the distribution of alloantibodies by gender, as shown in Table 3, alloantibodies belonging to different blood systems were identified in the female population. The most frequent were anti-D (28.7%), anti-E (14.7%), and anti-Kell (10.8%), while other clinically significant alloantibodies such as anti-Jka, anti-Jkb, anti-Fya, anti-Lea, anti-Leb, anti-M, anti-S, and anti-Dia were also detected. In men, a smaller number of alloantibodies were characterized, with anti-D (13.9%), anti-E (9.7%), and anti-Kell (8.3%) being the most prevalent.

**Table 3:** Distribution of alloantibodies according to gender detected in the 201 irregular antibody-positive patients, admitted to the Transfusion service (AGETRA) of Hospital de Clínicas of the Federal University of Uberlândia (HC-UFU/EBSERH), in the period from January 2019 to December 2020.

Blood group	Antibody type	Gender	
		Male	Female
		Total 72	Total 129
		n (%)	n (%)
Rh	Anti-D	10 (13.9)	37 (28.7)
	Anti-c	0 (0.0)	7 (5.4)
	Anti-C	5 (6.9)	9 (7.0)
	Anti-E	7 (9.7)	19 (14.7)
	Anti-e	1 (1.4)	1 (0.8)
Kell	Anti-Kell	6 (8.3)	14 (10.8)
Duffy	Anti-Fya	3 (4.1)	2 (1.5)
Kidd	Anti-Jka	3 (4.1)	4 (3.1)
	Anti-Jkb	1 (1.4)	1 (0.8)
Lewis	Anti-Lea	3 (4.1)	4 (3.1)
	Anti-Leb	0 (0.0)	2 (1.5)
MNS	Anti-M	0 (0.0)	9 (7.0)
	Anti-S	0 (0.0)	2 (1.5)
Diego	Anti-Dia	0 (0.0)	3 (2.3)
NI^a^	NI^a^	5 (2.9)	13 (10.1)

a Antibodies not identified by the RBC panel used.

Seventy-six patients (37.8%; 76/201) underwent erythrocyte immunophenotyping as shown in Table 4. The female gender (55.3%) was more prevalent than the male gender (44.7%); 31.6% of patients were transfused due to preoperative procedures and 26.3% due to malignancies. During the hospitalization period, 46.1% of patients received 1-3 transfusions, and 31.6% had a previous history of transfusion. Additionally, 39.5% of patients had undergone previous surgical procedures. The antigens identified in immunophenotyped patients were e (100%), c (86.8%), C (40.8%) and E (17.1%).

**Table 4:** Clinical, epidemiological, and laboratory characteristics of 76 irregular antibody-positive patients submitted to erythrocyte immunophenotyping evaluated by the transfusion service (AGETRA) of Hospital de Clínicas of the Federal University of Uberlândia (HC-UFU/EBSERH), from January 2019 to December 2020.

Characteristic	n (%)
**Gender**	
Male	34 (44.7)
Female	42 (55.3)
**Age (IQR**^a^**)**	53 (40-67)
**Ethnicity**	
White	35 (46.1)
Others	41 (53.9)
**ABO typing**	
A	32 (42.1)
B	12 (15.8)
AB	5 (6.6)
O	26 (34.2)
Indefinite	1 (1.3)
**RhD typing**	
Positive	48 (63.1)
Negative	27 (35.6)
Indefinite	1 (1.3)
**Erythrocyte immunophenotyping**	
C antigen	31 (40.8)
c antigen	66 (86.8)
E antigen	13 (17.1)
e antigen	76 (100)
**Clinical indication for transfusion**	
Preoperative	24 (31.6)
Anemia	4 (5.3)
Pregnancy/Birth	7 (9.2)
Kidney or Heart Disease	8 (10.5)
Hematologic disease	13 (17.1)
Malignancy^b^	20 (26.3)
**Number of transfusions received during hospitalization**	
>10	8 (10.5)
4 to 10	7 (9.2)
1 to 3	35 (46.1)
0	26 (34.2)
**Transfusion history**	
Yes	24 (31.6)
No	50 (65.8)
Uninformed	2 (2.6)
**Previous surgery**	
Yes	30 (39.5)
No	46 (60.5)
Uninformed	0 (0.0)
**Length of stay, in days (IQR)**	7 (1-21)
**Outcome 30 days after last transfusion**	
Discharge	72 (94.7)
Death	4 (5.3)

a Interquartile range.

b Solid tumor and/or oncohematologic disease.

In this study, complete data was retrieved for 14 out of 18 IAS-positive pregnant women, whose alloantibodies were characterized and are presented in Table 5. The average age of the patients was 27 years, and most were multiparous. Among the 14 patients, 12 (85.7%) had anti-D as the most prevalent antibody, 7 (7/14; 50%) had previous use of anti-Rh immunoglobulin (anti-RhIg), and blood type A negative was the most frequent (4/12; 33.3%). A history of previous transfusion was identified in only one pregnant woman. Notably, Patient 9, who was A positive, presented with anti-Lea alloantibody, an unusual profile for alloimmunization induced by isoimmunized pregnancy. Erythrocyte phenotyping was not performed for most of the pregnant women.

**Table 5:** Clinical and transfusion characteristics of irregular antibody-positive pregnant women evaluated by the transfusion service (AGETRA) of Hospital de Clínicas of the Federal University of Uberlândia (HC-UFU/EBSERH),from January 2019 to December 2020.

Patient	Alloantibodies	RhIg	Obstetrical history^a^ (n)	Age (years)	Clinical indication of transfusion	Transfusion history (n)	Previous surgery	Blood typing	Erythrocyte Phenotype
1	Anti-D	UD^b^	G2P2A0	25	HDN	No	No	B negative	–
2	Anti-E	No	G3P3A0	22	Cesarean iterative	No	No	B positive	–
3	Anti-D	UD	G3P2A1	18	HDN	No	Yes	A negative	–
4	Anti-D	Yes	G1P1A0	24	Postpartum bleed	Yes	Yes	O negative	c, e
5	Anti-D	Yes	G4P3A1	35	Ectopic pregnancy	No	Yes	O positive^c^	–
6	Anti-D	Yes	G4P3A1	22	Abortion	No	Yes	O negative	c, e
7	Anti-D, Anti-C	UD	G5P4A1	33	HDN	No	Yes	AB negative	c, e
8	Anti-D	Yes	G2P2A0	18	Puerperal bleeding	No	No	A negative	c, e
9	Anti-E, Anti-Lea	No	G3P3A0	33	Gestation high risk	No	Yes	A positive	–
10	Anti-D	Yes	G1P1A0	32	High risk bleeding	No	Yes	O negative	–
11	Anti-D	Yes	G2P1A1	24	Pregnancy	No	Yes	B negative	–
12	Anti-D, Anti-G	UD	G2P2A0	20	HDN (First pregnacy)	No	Yes	A negative	–
13	Anti-D	Yes	G2P1A1	31	Ectopic pregnancy	No	Yes	AB negative	–
14	Anti-D	UD	G2P2A0	38	High risk bleeding	No	Yes	A negative	–

RhIg: Rh immune globulin; HDN: Hemolytic Disease of the Newborn; UD: Unavailable Data –: Not performed.

a Number of Pregnancies.

b Viable births and Abortions.

c Weak agglutination RhD.

## Discussion

In this study the prevalence of positive IAS samples was 1.3%, a similar rate to that of Pereira Bueno et al., who reported a rate of 1.1%.^9^ Despite the advances in the safety of transfusion medicine, adverse effects such as alloimmunization, acute and late transfusion hemolytic reactions, and iron overload are still observed, conditions that are more prevalent among polytransfused patients specifically those with hematological/oncohematological diseases.^10^, ^11^ However, early recognition and proper management can prevent more severe outcomes for patients.

Due to miscegenation, a characteristic of the Brazilian population, there is a heterogeneity of phenotypic frequencies of blood systems, which explains the wide range of polymorphisms.^12^ Corroborating the data available in the literature, which report that blood groups A and O, both RhD positive, are the most frequent in Brazil, these systems were predominant in this research.^12^, ^13^, ^14^ Furthermore, alloimmunization was common in older patients. This fact was also reported by another study, in which over 30-year-old patients had a greater chance of developing alloimmunization with the risk increasing with age.^15^, ^16^, ^17^ Therefore, in addition to the transfusion history, age can also influence the positivity of IAS.

Regarding ethnicity, the distribution between whites and non-whites within this study was similar, which suggests that this is not a factor related to the development of alloimmunization. Gender, in another way, seems to be related to this adverse effect, since more women had positive IAS. Other studies have also shown a higher frequency of alloimmunization in female patients, which is explained by the gestational history and exposure to different antigens.^18^, ^19^, ^20^

The erythrocyte antigens detected in this study, D, E, e, C and c, are highly immunogenic. For this reason, their respective antibodies (anti-D, anti-E, anti-e, anti-C and anti-c) can induce acute or delayed post-transfusion hemolytic reactions and hemolytic disease of the newborn (HDN).^21^^,^^22^ The results presented confirm the high detection and significance of the Rh system, which is consistent with previous studies.^8^^,^^21^

The Rh system is one of the most polymorphic and immunogenic known in humans and represents the most significant cause of hemolytic transfusion reactions.^10^ The system has about 55 erythrocyte antigens, five of which are of greater clinical importance: D, E, e, C, and c.^3^ These antigens are glycoproteins important to the integrity of the erythrocyte membrane, with structural function and responsible for gas transport.^2^ Various polymorphisms in the proteins of this system make it more likely to induce intense immune responses.^23^ The Kell system, also considered one of the most immunogenic, has 36 antigens, with K and k being the most clinically important.^3^ The antibodies Anti-K and Anti-k, belong to the IgG class and are fully formed at birth, a relevant characteristic, particularly in pregnancy, since they can attack erythrocyte precursors of the fetus, resulting in severe anemic conditions.^14^^,^^24^ Together with the effects of the antibodies against the Rh system, they are closely related to severe acute or delayed hemolytic reactions, in addition to HDN.^2^^,^^14^^,^^25^

The distribution of alloantibodies varies according to the study population, however, the prevalence of Anti-D, Anti-E, and Anti-Kell demonstrated in this work, corroborate the data available in the literature, which show a higher prevalence of these antibodies in several countries, including Brazil.^10^^,^^19^^,^^26^ The literature also demonstrates the frequent identification of other systems in different populations, as is the case of Anti-E, Anti-D, and Anti-M in Chinese,^26^ and Anti-E, Anti-Lea, Anti-K and anti-D in North Americans.^27^ The high immunogenicity, individual immune response, dose, and frequency of transfusion are characteristics that increase the probability of irregular antibodies against blood systems such as Rh and Kell.^16^^,^^17^^,^^28^ In addition, immune response, dose, and frequency of transfusion are also characteristics that increase the probability of irregular antibodies developing.^28^

Several studies highlight the concern with patients at high risk of alloimmunization due to the need for multiple transfusions, for example, patients with hematological diseases such as sickle cell anemia and thalassemia, and oncohematological diseases, such as myelodysplastic syndromes, leukemias and lymphomas.^17^^,^^21^^,^^22^^,^^29^ These individuals are exposed to different erythrocyte antigens more and are therefore more likely to produce alloantibodies.^7^ Otherwise, in this study, transfusion was indicated mainly as part of preoperative procedures, which is justified by the profile of the population treated at the hospital. Although a limited number of studies have explored the characteristics of alloimmunized patients due to preoperative procedures, it is well known that phenotyping is not typically conducted during emergency transfusions. Furthermore, even in the absence of repeated transfusions, immunological effects can be induced. These effects can persist beyond their detectability and result in delayed transfusion reactions.^19^^,^^28^^,^^30^

Proper management of blood components, particularly with regards to the Rh and Kell blood systems, aims to prevent the co-occurrence of alloantibodies, as this association is often more frequent in patients who have received multiple transfusions and are therefore more likely to experience post-transfusion hemolytic reactions.^26^ Due to the immunogenicity of these two systems, the results of this study are consistent with the literature, as the most prevalent associations were found between Rh and Kell.^26^^,^^31^ However, the Rh system was the most frequently involved, likely due to the high genetic variability of RhD^23^ as demonstrated by Politou et al.^19^ The high frequencies of Anti-E and Anti-Kell, in addition to the data on associations, warrant special attention to these systems due to the high prevalence of Anti-D, Anti-E, and Anti-Kell antibodies in the population, particularly in women.

Regarding the formation of antibodies, the ‘unidentified’ antibodies that were detected and found relevant in this study may have been due to pan reactivity with autoantibodies. This class of antibodies can lead to a masking effect on the presence of alloantibodies, making patient management challenging.^10^^,^^17^ However, it is known that additional dilution and adsorption techniques can aid in the separation and identification of alloantibodies.^17^^,^^32^

The care aimed at preventing alloimmunization in women should primarily focus on pregnancies due to the higher frequency of alloimmunization in this population caused by exposure to different antigens.^10^^,^^14^^,^^20^ In the current study, a high frequency of antibodies against Rh system antigens was observed in correlation with gender and blood systems. This is due to the higher frequency of the Rh system in the Brazilian population.^16^^,^^33^ The MNS system was the third most common blood system with antibody development, and interestingly, alloantibodies from this system only appeared in women, which differs from other studies.^19^^,^^26^ Although not clinically significant, Anti-M is part of a complex blood system and was frequently detected in our study data.^2^^,^^14^ Finally, the proportion of alloimmunized men and women followed the pattern of 1.8% to 2.7% found in other studies.^19^^,^^34^^,^^35^

The epidemiological and clinical characteristics of immunophenotyped patients were evaluated thus identifying the most frequent erythrocyte antigens. The highest frequencies were the e, c, and C antigens. This phenotypic screening is essential for enhancing transfusion safety and preventing future alloimmunization. Therefore, measures such as performing extended phenotyping can help prevent and reduce this condition.^10^^,^^21^^,^^22^^,^^36^^,^^37^

Concerning alloimmunized pregnant women, it was observed that, despite the various clinical indications for transfusion, most were multiparous, supporting the possibility of maternal-fetal sensitization, as demonstrated in other studies.^21^^,^^28^ The most frequently found antibodies were of the Rh blood system, indicating possible alloimmunization induced by isoimmunized pregnancies.^20^

The use of human anti-D immunoglobulin in pregnant women with RhD antigen incompatibility with their partner is one of the methods employed to prevent HDN and maternal alloimmunization during prenatal care.^18^ In this study, it was not possible to distinguish whether the production of alloantibodies in pregnant women occurred due to sensitization in previous pregnancies, the use of the medication (Rho(D) immune globulin), or a history of transfusions outside HC-UFU/EBSERH, due to the absence of prior negative results and previous medical records. Nevertheless, it is undeniable that the rates of alloimmunization in pregnant women who use immunoglobulin during prenatal care are reduced.^38^

The main feature of the present study was the utilization of a retrospective design, limiting patient analysis by not providing longitudinal follow-up information about their outcomes. Furthermore, being a single-center study, it was not possible to verify previous transfusions in other health facilities, which could directly impact the laboratory results analyzed. Additionally, only the initial positive sample from each patient was evaluated in this study, potentially limiting the identification of new alloantibodies produced after the patient's exposure to transfusion and other antigenic stimuli. Nevertheless, the impossibility of following patients over time, as in a prospective study, justifies this methodological decision. At the national level, it is important to acknowledge that Brazil is a developing country, and certain regions, such as the one in this study, may lack the necessary resources to perform phenotyping for all patients at the local hospital.

## Conclusions

This study found that the majority of patients who received transfusions were female and underwent preoperative procedures. Alloantibodies from the Rh and Kell blood systems, which are highly immunogenic, were frequently detected. Therefore, the screening and characterization of alloantibodies, along with erythrocyte immunophenotyping, are necessary to better understand the alloimmunized population. This approach can improve the safety and efficacy of transfusion therapy, reducing the risk of serious reactions. This is particularly important for oncohematological, chronic renal, transplanted, and polytransfused patients.

## Conflicts of interest

The authors declare no conflicts of interest.
